# *N*‐Glycoproteomics of the Apicomplexan Parasite *Toxoplasma gondii*

**DOI:** 10.1002/pmic.202400239

**Published:** 2025-03-12

**Authors:** Vanessa Horn, Patricia Zarnovican, Birgit Tiemann, Andreas Pich, Hans Bakker, Françoise H. Routier

**Affiliations:** ^1^ Department of Clinical Biochemistry OE4340 Hannover Medical School Hannover Germany; ^2^ Department of Toxicology OE5340 Hannover Medical School Hannover Germany

**Keywords:** apicomplexa, BC2L‐A lectin, *N*‐glycosylation, parasite

## Abstract

Protein *N*‐glycosylation influences protein folding, stability, and trafficking, and has prominent functions in cell–cell adhesion and recognition. For the parasite *Toxoplasma gondii*, *N*‐glycosylation of proteins is crucial for initial adhesion to host cells, parasite motility, and consequently, its ability to invade host cells. However, the glycoproteome of *T. gondii* remains largely unknown. In this study, we used the α‐mannose‐specific *Burkholderia cenocepacia* lectin A (BC2L‐A) to enrich glycopeptides from *T. gondii* tachyzoites and analysed them by tandem mass spectrometry. The data enable the identification of over 100 *N*‐glycoproteins with the glycosylation site(s) and the composition of the *N*‐glycans at each site. *T. gondii* glycoproteins include known virulence factors, vaccine candidates as well as numerous uncharacterised proteins. These data provide ground knowledge to deepen our understanding of the role of glycoproteins in invasion and assist the rational design of vaccines.

**Abbreviations**
Asn: asparagine
BC2L‐A: *Burkholderia cenocepacia* lectin A
CLPTM1: cleft lip and palate transmembrane protein 1
CLPTM1L: cleft lip and palate transmembrane protein 1 like
ConA: concavalin A
ER: endoplasmic reticulum
GAP50: gliding associated protein 50
GlcNAc: *N*‐acetylglucosamine
GPI: glycosylphosphatidylinositol
HCD: higher‐energy collisional dissociation
Hex: hexose
HexNAc: *N*‐acetylhexosamine
Hsp70: heat shock protein 70
hyperLOPIT: hyperplexed localisation of organelle proteins by isotope tagging
IMC: inner membrane complex
Man: mannose
ppGalNAc‐T: polypeptide N‐acetylgalactosaminyltransferase
OST: oligosaccharyltransferase
Ser: serine
Thr: threonine

1

*Toxoplasma gondii* is an obligate intracellular apicomplexan parasite that may infect all warm‐blooded animals. Toxoplasmosis, the infection caused by *T. gondii*, is usually asymptomatic or is a mild illness with flu‐like symptoms. However, infection acquired during pregnancy may cause abortion or foetus malformations. Tissue cysts or oocysts enter the body through ingestion and transform into rapidly dividing tachyzoites that disseminate into the bloodstream and actively invade host cells. Because of the inflammatory response, the tachyzoites transform into slowly replicating bradyzoites, which encysts and persist lifelong in brain, skeletal muscle, and cardiac muscle. In immunocompromised individuals, especially in HIV patients, bradyzoites may retransformed into tachyzoites and lead to severe or even lethal infections [1].

*T. gondii* harbours a specialized endomembrane referred to as the inner membrane complex (IMC) placed directly beneath the plasma membrane. The IMC separates most of the plasma membrane from the cytoplasm and hinders the processes of endocytosis and exocytosis. The IMC is however discontinuous at the anterior end of the tachyzoite, where regulated secretion from specialised organelles occurs through the apical complex. Protein secretion from the micronemes, rhoptries and dense granules is crucial for motility, host cell invasion and the subversion of host cellular functions [2, 3]. These proteins transit via endoplasmic reticulum (ER) and Golgi apparatus, where they can be substituted with glycans.

*T. gondii* proteins can be modified by *N*‐, *O*‐, *C*‐glycans, and GPI‐anchors [4, 5, 6, 7]. *T. gondii* genome encodes ALG enzymes required for the biosynthesis of a *N‐*glycan precursor and a functional oligosaccharyltransferase (OST) responsible for the transfer to proteins [8, 9, 10, 11]. According to the predicted set of ALG enzymes, *T. gondii* would synthesise a Glc_3_Man_5_GlcNAc_2_‐PP‐Dol precursor. However, analysis of *N*‐glycans released from *T. gondii* proteins suggested Man_6_GlcNAc_2‐_ *N*‐glycans with 0, 1 or 2 glucose residues arising from a Glc_3_Man_6_GlcNAc_2‐_PP‐Dol precursor [9]. The authors proposed the transfer of an additional α1,2‐mannose by ALG11 [9]. These structures are in agreement with the presence of the ER glucosidase I and II and the absence of further genes encoding enzymes involved in trimming or maturation of *N*‐glycans [9, 12]. The importance of glycosylation for *T. gondii* is suggested by the low fitness score associated with the deletion of many glycosyltransferase genes [9, 13]. Moreover, tachyzoites treated with the *N*‐glycosylation inhibitor tunicamycin lost their motility and ability to invade host cells [14]. This phenotype could be partly explained by the requirement of the gliding associated protein 50 (GAP50) *N*‐glycosylation for its trafficking and interaction with key proteins of the glideosome [15].

Despite the importance of glycosylation, the glycoproteome of *T. gondii* is still largely unknown. An early proteomic study by Luo et al. identified up to 132 proteins potentially *N*‐ or *O*‐glycosylated that were isolated from *T. gondii* extract by serial lectin affinity chromatography [16]. More recently, Nazarova et al. identified 89 proteins using metabolic labelling of tachyzoites with GlcNAc analogues coupled to click chemistry. However, the vast majority of labelled proteins were *O*‐glycosylated [17]. We recently described the α‐mannose‐specific *Burkholderia cenocepacia* lectin A (BC2L‐A) as a tool for the identification of *O*‐mannosylated and *C*‐mannosylated proteins [18]. In this study, we used this lectin for the capture of *N*‐glycopeptides obtained by protease digestion of tachyzoites total extract and analysed them by LC‐MS/MS (see Material and Methods in Supporting Information). This glycopeptidomics approach enabled the identification of 133 glycoproteins including the description of the *N*‐glycosylation sites and composition of the *N*‐glycans.

ConA is a broadly selective lectin that preferentially recognizes the conserved trimannoside core of *N*‐glycans [19]. In contrast, *B. cenocepacia* BC2L‐A lectin binds a single α‐mannose residue with high specificity and affinity [20]. Considering the reported structures of *T. gondii N*‐glycans [9], we compared the ability of both lectins to enrich glycopeptides from a complex tryptic digest of tachyzoites extract. Both lectins require calcium ions for binding. However, since the two calcium ions present in the BC2L‐A binding site are weakly bound [21], a low concentration of EDTA can be used for the elution of the glycopeptides. In the case of ConA, elution with EDTA is inefficient and the glycopeptides were eluted with methyl‐α‐D‐mannose [18]. Obtained glycopeptides were analysed by LC‐MS/MS with HCD fragmentation and analysis of MS/MS spectra using FragPipe [22]. *N*‐Glycosylated peptides were searched with *N*‐glycan open search settings with search restricted to the *N*‐glycosylation consensus sequence (Asn‐X‐Thr/Ser where X is any amino‐acid except for proline). Since *T. gondii* is an obligate intracellular parasite and contamination with host cells is possible, we analysed the data with a combined *T. gondii*/human database to ascertain that selected peptides are unique to *T. gondii*. The MS/MS spectra usually display ions corresponding to glycosidic bond fragmentation and are dominated by the fragment ion of the intact peptide with one HexNAc residue. The *N*‐glycan composition can be deduced from the mass difference between the precursor ion and the peptide fragment ion. Lower abundance peptide backbone fragments are observable and support the peptide sequence assignment. All peptides presented in Tables S1 and S2 were manually validated. As inclusion criteria, the HCD spectrum had to display a minimum of four y‐ or b‐ions, fragment ion peaks for the peptide without *N*‐glycan, the peptide with 1 GlcNAc residue, as well as GlcNAc oxonium ion(s).

The tryptic peptide GVNVTIDR of the dense granule protein GRA57 (TGRH88_059610) is provided as an example (Figure 1). The mass offset between [M+2H]^2+^ precursor ion at *m*/*z* 1288.534 and unglycosylated peptide fragment ion (*m*/*z* 873.478) reveals the presence of a Hex_8_HexNAc_2_ *N*‐glycan. The fragment ion of the intact peptide with one HexNAc residue at *m*/*z* 1076.56 dominates the spectrum. The spectrum displays oxonium ions at *m*/*z* 204.08, 366.13 and 528.18 corresponding to HexNAc, HexHexNAc and Hex_2_HexNAc, respectively. The peptide sequence is inferred from low‐intensity b‐ and y‐ion peptide backbone fragments displayed in the magnified view of the HCD MS/MS spectrum (Figure 1).

**FIGURE 1:**
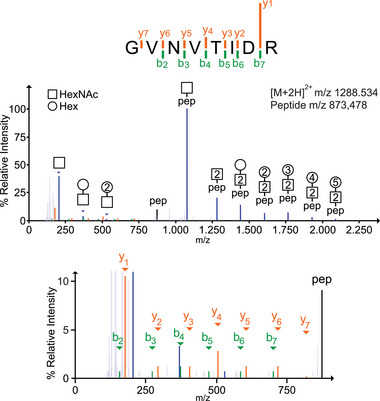
Example of glycopeptide HCD tandem mass spectrum. Spectrum of the peptide GVNVTDR modified with Hex_8_HexNAc_2_; precursor ion [M+2H]^2+^ at m/z 1288.534. Glycosidic bond fragmentation defines the sequence of the glycan. A magnified view of the area between 100 and 900 displays the lower intensity b‐ and y‐ion peptide backbone fragments, which delineate the peptide sequence.

In this first experiment, we identified 102 peptides originating from 84 *N*‐glycoproteins using BC2L‐A and 19 peptides from 18 glycoproteins after ConA affinity chromatography (Table S1). The BCL2‐A lectin appears superior to ConA for the capture of *N*‐glycans of the oligomannose type and was selected for further experiments. This lectin would be well suited for glycoproteomic studies of organisms (e.g., protists, fungi) that do not synthesize complex‐type *N*‐glycans.

To increase glycopeptide coverage, we digested tachyzoite extracts with trypsin or a combination of trypsin and AspN or trypsin and GluC. The glycopeptides were enriched by BCL2‐A affinity and analysed by tandem mass spectrometry as described in Supporting Information. The density of *N*‐glycosylation sites is lower in *T. gondii* than in other eukaryotes, since Asn is encoded by AAT or AAC and *T. gondii* coding sequences have a 41% AT content [12, 23]. Moreover, there seems to be no positive selection for putative *N*‐glycan sites in *T. gondii* secreted proteins in comparison to cytosolic proteins in accordance with the absence of quality control of glycoprotein folding in this organism [6, 12]. Nonetheless, our analyses identified 178 glycopeptides belonging to 133 *N*‐glycoproteins, which corresponds to an average of 1.3 *N*‐glycan/identified glycoprotein (Table S2).

In addition, we searched for *C*‐mannosylated peptides, which are known ligands of BCL2‐A [18]. *C*‐mannosylated proteins are involved in parasite adhesion, motility and virulence but, with the exception of the micromenal protein MIC2, remain unidentified [4, 24, 25]. We confirmed the *C*‐mannosylation of the thrombospondin repeats of endogenous MIC2 (TGRH88_037170). The HCD MS‐MS spectra of TSR2, 4 and 6 presented in Figure S1, clearly show the presence of a hexose on the tryptophan residues. Moreover, we found evidence for *C*‐mannosylation of the thrombospondin type 1 domain containing protein (TGRH88_022040) (Figure S2). Using MIC2‐deficient parasites and/or enzymatically removing *N*‐glycans might enable the identification of further *C*‐mannosylated proteins.

About one‐fourth of identified *N*‐glycoproteins are predicted proteins of unknown functions (Table S2). Many others are annotated based on sequence conservation but have not been characterised. Predictions of signal peptide and transmembrane domain(s) were carried out using the algorithms SignalP ‐ 6.0 and DeepTMHMM [26, 27]. Numerous identified proteins were predicted to lack both a signal peptide and a transmembrane domain. The signal sequences of *T. gondii* proteins are often atypical and known to be difficult to predict. Nevertheless, since *N*‐glycosylation occurs in the lumen of the ER, identified glycoproteins are expected to transit via this organelle. For example, uroporphyrinogen decarboxylase and IMC19 protein have no predicted signal peptide or transmembrane domain but were shown to localise to the apicoplast and IMC, respectively [28, 29]. In addition, we retrieved the hyperplexed localisation of organelle proteins by isotope tagging (hyperLOPIT) and the CRISPR phenotype score from the ToxoDB database [30] (Table S2). This score reflects the fitness that a specific gene confers to the parasite for infection of human fibroblast. Although we carried our experiment with a type I RH88 strain, the predicted localisation and fitness score relate to the corresponding gene in a type II ME49 strain and type I GT1 strain, respectively.

Amongst the 133 glycoproteins, 44 were predicted to principally localise to the ER and Golgi, 29 to the micronemes, rhoptries or dense granules, 16 at the plasma membrane or IMC and 16 to the apicoplast. Surprisingly, we also identified six proteins localised to the cytosol or nucleus by hyperLOPIT. The observed molecular ions and fragments ions displayed in the MS/MS spectra clearly indicate the presence of *N*‐glycans on these proteins (Figure S3). It would be interesting to reassess their localisation with additional methods. To date, the membrane‐bound OST is the only enzyme associated with *N*‐glycosylation of proteins in prokaryotes and eukaryotes. This enzyme acts in the periplasm or lumen of the ER to modify secreted and membrane‐bound proteins. In *T. gondii*, *O*‐fucosylation of numerous nuclear or cytoplasmic proteins by the glycosyltransferase spindly has been described. Several cytoplasmic glycosyltransferases also modify the protein Skp1 with an unusual O‐glycan [5, 31–33]. Currently, we cannot exclude the existence of a novel cytoplasmic *N*‐glycosylation machinery.

Although Asn‐X‐Thr sequons are less frequent than Asn‐X‐Ser sequons in *T. gondii* genome [12], ∼83% of the occupied *N*‐glycosylation sites contained Thr rather than Ser. Such a marked preference for Asn‐X‐Thr has also been reported for the apicomplexan *Cryptosporidium parvum* [34] and likely reflects the preference of OST for sequon with Thr [35]. Moreover, *T. gondii* OST clearly differs from the single subunit OSTs of bacteria or *Trypanosoma brucei* and does not favour sequons with an acidic residue at position −2 [35, 36] as displayed in Figure 2A.

**FIGURE 2:**
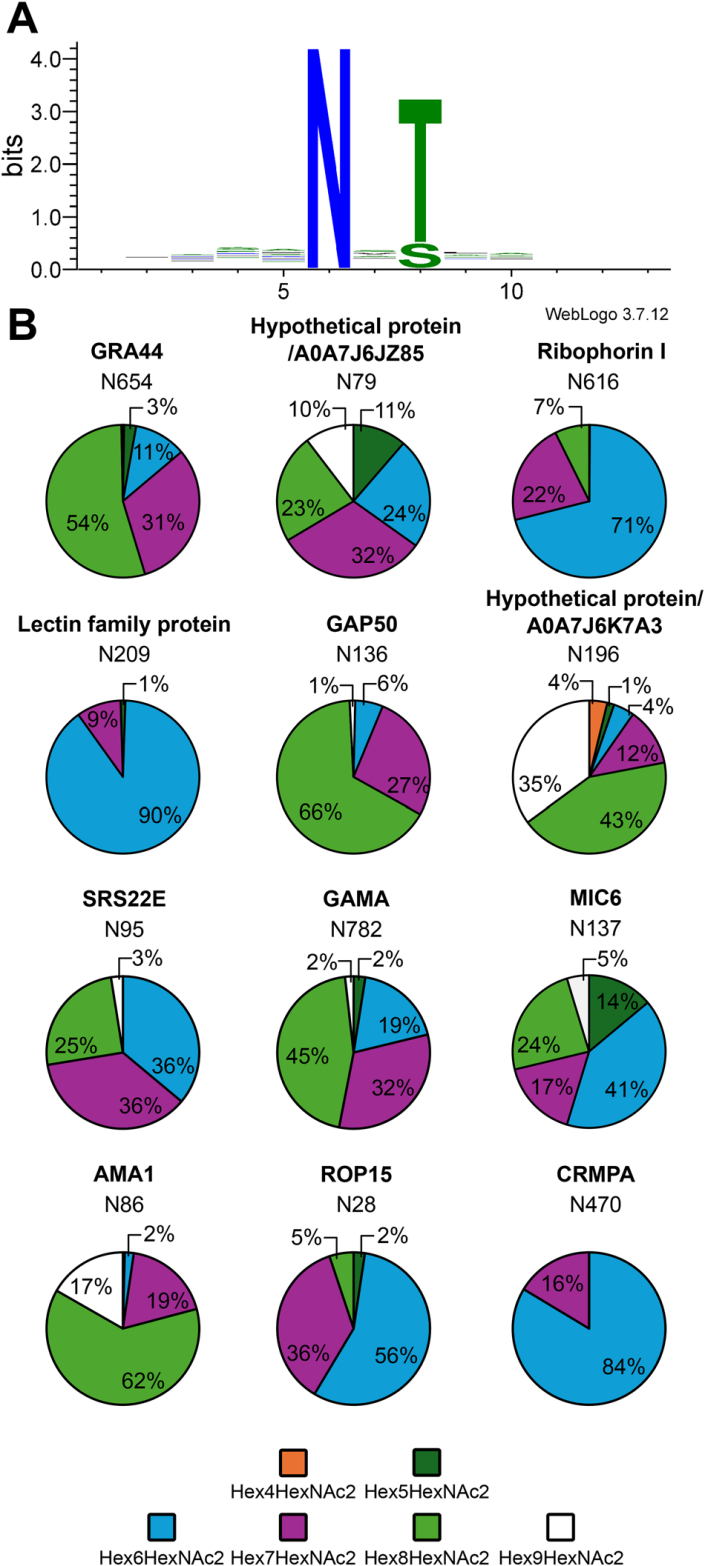
Analysis of identified glycopeptides. (A) The Web‐Logo generated with all glycopeptides sequences identified in this study underlines the high occupancy of sequons with threonine; (B) Glycoforms distribution of peptides from various proteins. The pie charts were generated by integration of the molecular ions corresponding to the different glycoforms of a specific peptide.

Glycoforms identified from the glycopeptide mass and the MS/MS spectrum are presented in Table S2. In agreement with the study of Gas‐Pascual et al. the proteins carried *N*‐glycans with mainly 6, 7 or 8 hexoses and two *N*‐acetylhexosamines, which likely correspond to Man_6_GlcNAc_2_, GlcMan_6_GlcNAc_2_ and Glc_2_Man_6_GlcNAc_2_ [9]. The areas of the precursor molecular ions corresponding to the different peptide glycoforms can be used to estimate their distribution. This evaluation was carried out for a few proteins with different subcellular localisation and is presented in Figure 2B and Table S3. This quantification of the site‐specific glycoform distribution illustrates the predominance of Hex_6_HexNAc_2_, Hex_7_HexNAc_2_ and Hex_8_HexNAc_2_ *N*‐glycans. According to the structures described in *T. gondii*, Hex_7_HexNAc_2_ and Hex_6_HexNAc_2_ arise from the processing of Hex_8_HexNAc_2_ by glucosidase II [9]. As expected, the extent of processing is protein‐ and site‐specific and likely reflects the accessibility of the *N*‐glycan to glycosidases. Hex_3_HexNAc_2_, Hex_4_HexNAc_2_ and Hex_5_HexNAc_2_ *N*‐glycans are rare and of low abundance and might arise from degradation during the sample preparation. Finally, Hex_9_HexNAc_2_, identified in a few glycopeptides, likely correspond to unprocessed Glc_3_Man_6_GlcNAc_2_ *N*‐glycans.

Approximately one‐third of the identified glycoproteins were predicted to reside in the ER or Golgi and include proteins potentially involved in glycosylation, folding or vesicular transport (Table 1 and Table S2). The catalytic subunit STT3 of *T. gondii* (TGRH88_075680) OST was found to carry *N*‐glycans in its C‐terminal luminal domain at Asn590, Asn597 or Asn601, and Asn700. Since the peptide backbone fragments tend to lose their glycan upon HCD, it is not possible to define which of the two putative *N*‐glycosylation sites is occupied in the peptide TVLVDN_597_NTWN_601_NTHIATVGLALSSNEEK. However, Asn597 is placed downstream of the sequon binding motif WWD_580_ and is part of the conserved motif DNNTWN [35, 37]. Ramirez et al. showed that the Thr residue and preceding Asn in this motif are involved in the recognition of the GlcNAcβ1,4GlcNAc core, whereas the first Asn is involved in the binding of a nonreactive peptide [37]. *N*‐glycosylation of the Asn597 residue is therefore unlikely since it would prevent peptide binding by STT3 and the subsequent transfer of the oligosaccharide [37]. In addition to STT3, the type II transmembrane protein Ost1/Ribophorin1 (TGRH88_036440) was found to be *N*‐glycosylated at Asn616 and Asn676. The precise role of this noncatalytic OST subunit is still unclear. *T. gondii* genome also encodes Wbp1/OST48 and Ost2/DAD1 subunits [34] involved in recognition of the terminal glucose residues of the precursor [37]. The genes encoding these OST subunits all confer in vivo fitness to tachyzoites and their deletion is associated with a low fitness score (Stt3: ‐3.45; Ost1: ‐3.10; Wbp1: ‐4.25; Ost2: ‐3.02) [13] reflecting the importance of *N*‐glycosylation.

**TABLE 1:** Examples of glycoproteins.

Protein	Gene	Peptide	Asn	HyperLOPIT localisation	Main glycoform(s)
STT3	TGRH88_075680	IMSWWDYGYQATAMGNR TVLVDNNTWNNTHIATVGLALSSNEEK FANVTGGFDFAR	590 601 700	ER	Hex7HexNAc2 Hex6HexNAc2 Hex6HexNAc2
Ost1	TGRH88_036440	RAFSLEVFDQIGNVSSTR YTLNLTLAPPFR	616 676	ER	Hex6HexNAc2 Hex6HexNAc2
GPI EtNP‐T	TGRH88_044580	GGSLDAASTPAVNR	501	ER	Hex6HexNAc2
CLPTM1L	TGRH88_034270	VFNATYTYDR	228	ER	Hex6HexNAc2
CLPTM1L	TGRH88_003160	NNTTLYVHVR	143	ER	Hex6HexNAc2
ppGalNAc‐T2	TGRH88_071270	DASLPGQAASANATGR	556	Golgi	Hex6HexNAc2/ Hex7HexNAc2
PPI	TGRH88_000250	NHNGTGGHSIYGR	85	ER2	Hex6HexNAc2
Oxidoreductin	TGRH88_057000	RNVTQATGVYV	236	ER	Hex6HexNAc2
Hsp70	TGRH88_046410	LDLPTNTTATK DVGTHLNGDEAMATGAAFIAANSTATFR	408 610	ER2	Hex6HexNAc2 Hex8HexNAc2
SRS20A	TGRH88_026130	VTGNSSVEFK NGTLSLEVNPSKK	56 176	PM peripheral 1	Hex6HexNAc2 Hex9HexNAc2
SRS20C	TGRH88_026150	VTNLPKNETTFCYK	257	PM peripheral 1	Hex8HexNAc2
SRS22E	TGRH88_032060	IETCAPDKPISFNVTEAGQSILFK	95	/	Hex6HexNAc2/ Hex7HexNAc2
SRS29C	TGRH88_077470	CSYTENSTLPK YNCTVPVQLGGEDPSEGSRPGGGSGGGKR	202 302	PM peripheral 1	Hex6HexNAc2 Hex6HexNAc2
SRS54	TGRH88_054600	IPPGDFPEVPVNLTLSCQQK	323	PM peripheral 1	Hex7HexNAc2
SRS protein	TGRH88_046180	HKNNNTLCFVDVTVK	152	/	Hex6HexNAc2
MIC4	TGRH88_022630	ITPAGDDVSANVTSSEPAK	55	Micronemes	Hex8HexNAc2
MIC6	TGRH88_058880	ISLDGTGNVTCIVR	137	Micronemes	Hex6HexNAc2
GAMA	TGRH88_028570	MNNETVLYEPDTEIIEK DLAEFYNK TMNSEGVISDGLQSQLPVNHTR	699 762 782	Micronemes	Hex7HexNAc2 Hex8HexNAc2 Hex8HexNAc2
AMA1	TGRH88_073680	FNLTHHHQSGIYVDLGQDK	86	Micronemes	Hex8HexNAc2
SUB1	TGRH88_034960	AQGTIVEPDHLVQSVNTSSK	212	Micronemes	Hex6HexNAc2
ROM5	TGRH88_019690	KTYNAVLGNTTTPAAPSAAELAQQTR	821	PM‐integral	Hex6HexNAc2
GAP50	TGRH88_058240	VAANEHISFIASPGSNFLGGVSSLNDTR NYTSEALR	101 136	IMC	Hex7HexNAc2 Hex8HexNAc2
CRMPA	TGRH88_069420	NITDTAKLPDNILGR	470	Micronemes	Hex8HexNAc2
CRMPB	TGRH88_015880	ANDTSVHQYDR	6453	Micronemes	Hex6HexNAc2
RDF1	TGRH88_061300	DIVLDNSTIR	1713	Micronemes	Hex6HexNAc2
CARP	TGRH88_001930	ENSSTAVGSIPHFSALPESEWDYK NLTNVYMNA	102 157	Rhoptries 2	Hex6HexNAc2 Hex6HexNAc2
GRA16	TGRH88_022180	ASEGNASVNQTSPAASYPR	330	Dense granules	Hex8HexNAc2
GRA44	TGRH88_047510	NTTTAFITSGAAVR	654	Dense granules	Hex8HexNAc2

Abbreviations: AMA1, apical membrane antigen 1; CARP, carbonic anhydrase‐related protein; CLPTM1L, cleft lip and palate transmembrane protein 1 like; CRMPA, cysteine repeat modular protein CRMPA; CRMPB, cysteine repeat modular protein CRMPB; GAMA, glycosylphosphatidylinositol‐anchored micronemal antigen; GPI EtNP‐T, GPI‐ethanolamine phosphate transferase; Hsp70, heat shock protein 70/DnaK family protein; OST1, Oligosaccharyltransferase subunit 1/Ribophorin 1; ppGalNAc‐T2, polypeptide *N*‐acetylgalactosaminyltransferase; PPI, peptidyl–prolyl *cis*‐*trans* isomerase; RDF1, rhoptry discharge factor RDF1 (previously named MIC15); ROM5, rhomboid protease ROM5; SRS, GPI‐anchored surface antigen related sequence; STT3, Oligosaccharyltransferase catalytic subunit; SUB1, serine protease SUB1.

*Toxoplasma* expresses proteins involved in *N*‐glycan‐independent quality control of protein folding such as chaperones, protein disulfide isomerase (PDI) and peptidyl–prolyl *cis*‐*trans* isomerase [6]. Here, several proteins potentially involved in protein folding such as a putative peptidyl–prolyl *cis*‐*trans* isomerase (TGRH88_000250), ER oxidoreductin (TGRH88_057000), or a putative heat shock protein 70 (Hsp70) family member (TGRH88_046410) were shown to be *N*‐glycosylated (Table 1). Hsp70 chaperones prevent protein aggregation by recognizing hydrophobic stretches exposed on unfolded protein and contain an *N*‐terminal ATPase domain and a *C*‐terminal substrate binding domain. *N*‐glycosylation sequons tend to be disfavoured at protein–protein interfaces and are rare in ER Hsp70 chaperones [38]. The *T. gondii* putative Hsp70 (TGRH88_046410), annotated as DnaK family protein, exhibits a *C*‐terminal REEL sequence for retrieval to the ER and is *N*‐glycosylated at Asn270 and Asn472. A homologue of this protein is found in several alveolata, oomycota and in plants (Figure S4). The second *N*‐glycosylation site, situated at the end of the ATPase domain, seems to be conserved (Figure S4) except in *Theileria*, *Babesia* or *Plasmodium* (Figure S4). These apicomplexan parasites synthesise no or very short N‐glycans consisting of two GlcNAc residues [12].

Using purified protein, Fauquenoy et al. identified *N*‐glycans on three of the four putative *N*‐glycosylation sites of GAP50. Moreover, they demonstrated that the absence of a single *N*‐glycan at Asn101 or Asn136 impaired the trafficking of GAP50 to the IMC. The mutated proteins were retained in the ER and were unable to interact with the glideosome [15]. We confirmed *N*‐glycosylation of GAP50 at Asn101, Asn136 and Asn228 and identified an additional *N*‐glycan at Asn302. Interestingly, MIC4 carries a *N*‐glycan on Asn55, a site that directly precedes the microneme protein protease 3 (MPP3) cleaving site (NVT/SS) [39]. The protein is processed after release from the micronemes and would thus be devoid of *N*‐glycan when exposed at the parasite surface. It is therefore tempting to speculate that as for GAP50, the *N*‐glycan of MIC4 is required for protein folding and/or trafficking.

Finally, it should be underlined that several of the identified glycoproteins (e.g., MIC4, MIC6, AMA1, ROM5, SRS29C, MIC16) are immunogenic and potential vaccine candidates [40, 41, 42]. Glycosylation can greatly influence the conformational integrity and immunogenicity of a protein. Glycans often shield conserved protein epitopes but can also be part of the epitopes. Knowing and modulating the glycosylation of a protein is hence crucial for the rational design of vaccines [43].

## Conflicts of Interest

The authors have declared no conflict of interest.

## Supporting information



Supporting information

Supporting information

Supporting information

Supporting information

Supporting information

## Data Availability

Data have been deposited to GlycoPost [44] and are available at https://glycopost.glycosmos.org/preview/87699698867925365181dd with the PIN code 5686.
