# Supplementary material for: N‐Glycoproteomics of the Apicomplexan Parasite Toxoplasma gondii
Source: Proteomics. 2025 Mar 12;25(8):e202400239. doi: 10.1002/pmic.202400239 (PMC12019905; doi:10.1002/pmic.202400239)
Supplement: Supplementary file 1 — Supporting information [file PMIC-25-e202400239-s002.docx]

***Material and methods***

***T. gondii culture***

*T. gondii* strain RHΔku80Δhxgprt (Fox, Ristuccia, Gigley, & Bzik, 2009) was maintained by serial passage on monolayers of human foreskin fibroblasts (ATCC® SCRC-1041^TM^) grown at 37°C and 5% CO_2_ in Dulbecco’s modified Eagle’s medium (DMEM)/ F12 (1:1) containing 5% heat-inactivated fetal bovine serum (BioChrom), 100 U/mL penicillin and 100 µg/mL streptomycin (Life technologies). Freshly egressed tachyzoites were harvested by centrifugation at 1000 g for 5 minutes, and washed once with PBS. Harvested tachyzoites pellets were frozen at -80°C until further use.

***Protein extraction and digest***

For protein extraction, tachyzoites from nine T125 flasks were resuspended in 1 mL *Rapi*Gest SF (1 mg/mL) (Waters) in 50 mM ammonium bicarbonate and incubated on ice for 40 minutes. The lysed tachyzoites were sonicated and the sample centrifuged at 1000g for 10 minutes. To reduce protein disulfide bonds, 5 mM DTT was added to the *Rapi*Gest SF supernatant and incubated for 15 minutes at 72°C. After the addition of 10 mM Iodoacetamide, samples were incubated for 30 minutes at room temperature. Subsequently, 5 mM DTT and 25 µg Trypsin Gold (Promega) were added and the samples were incubated overnight at 37°C. In some experiments, AspN (400 ng) (Promega) or GluC (2 µg) (Promega) was added to the trypsin digest and the sample was further incubated for 5 hours at 37°C. The samples were acidified using TFA, loaded on equilibrated Sep-Pak C18 (50 mg) reversed phase cartridges (Waters). The columns were washed with 0.1% Formic acid and samples eluted with 50% acetonitrile were medium-dried in a Savant DNA_120_ SpeedVac concentrator without heat (Thermo Scientific).

***Preparation of Lectin Columns***

BC2L-A was produced in *E. coli* and purified as described previously (Hütte et al., 2022). ConA was purchased from Sigma. The lectins were coupled to a 1 mL HiTrap NHS-activated HP column (Cytiva) according to the manufacturer’s instructions.

***Lectin affinity purification***

Dried samples were resuspended in 1 ml BC2L-A buffer A (20 mM Tris-HCl pH 7.5, 150 mM NaCl, 2 mM CaCl_2_) or 1 ml ConA buffer A (20 mM Tris-HCl pH 7.5, 150 mM NaCl, 1 mM CaCl_2_ and 1 mM MnCl_2_), passed through a 0,22 µm filter (Merck). Samples were loaded onto an equilibrated BC2L-A/ConA lectin column using Äkta Pure FPLC chromatography system (Cytiva Life Science). After 5-30 column volumes (CV) wash with BC2L-A/ ConA buffer A, the peptides were eluted with BC2L-A buffer B (20 mM Tris-HCl pH 7.5, 150 mM NaCl, 20 mM EDTA) or ConA buffer B (20 mM Tris-HCl pH 7.5, 150 mM NaCl, 1 mM CaCl_2_ and 1 mM MnCl_2_, 0.1 M methyl α-D-mannoside). Peptide elution was followed by measuring the absorbance at 205 nm and 280 nm. Eluted peptides were collected, acidified with TFA and applied to equilibrated Empore C18 solid phase extraction cartridge (CDS analytical). The cartridges were washed with 2 mL 0.1% formic acid and the peptides eluted with 500 µL 50% acetonitrile and dried in a Savant DNA_120_ SpeedVac concentrator without heat (Thermo Scientific).

***MS measurement and Data processing***

LC-MS/MS measurements were carried out on a Thermo Fisher Scientific nano-flow UltiMate 3000 RSLCnano LC-system coupled to an Orbitrap Exploris™ 240 mass spectrometer. The LC was equipped with a trapping column (3 µm C18 particle, 2 cm length, 75 µm ID, Acclaim PepMap, Thermo Scientific) and a 50 cm µPAC™(Thermo Scientific) analytical column. Peptide mixtures were injected, enriched and desalted on the trapping column at a flow rate of 6 µL/min with 0.1% TFA for 5 min. The trapping column was switched online with the analytical column and peptides were eluted with a multi-step binary gradient: linear gradient of buffer B (80% ACN, 0.1% formic acid) in buffer A (0.1% formic acid) from 4% to 25% in 30 min, 25% to 50% in 10 min, 50% to 90% in 5 min and 10 min at 90% B. The column was reconditioned to 4% B in 15 min. The Flow rate was 500 nL/min and the column temperature was set to 35°C. The RSLC system was coupled online via a Nano Spray Source II (Thermo Scientific) to an Orbitrap Exploris 240 mass spectrometer. Metal-coated fused-silica emitters (SilicaTip, 10 µm i.d., New Objectives) and a voltage of 2.1 kV were used for the electrospray. Overview scans were acquired at a resolution of 120k in a mass range of m/z 300-1500. Precursor ions of charges two or higher and a minimum intensity of 4000 counts were selected for HCD fragmentation with a normalized collision energy of 30%. Active exclusion was set to 70 s within a mass window of 10 ppm of the specific m/z value. MS2 spectra were recorded with an isolation window of 1.6 at a resolution of 15k. Maximum injection time was set to 100 ms, first mass was set to 110 and a normalized automatic gain control (AGC) target was of 100% was used.

Raw MS data were processed using FragPipe (version 21.2) (Kong, Leprevost, Avtonomov, Mellacheruvu, & Nesvizhskii, 2017). Peptide spectrum matches were made with the UniProt *T. gondii* RH strain database (UP000557509) and human database (UP000005640). Peptide fragment ions b, y and precursor mass were assigned with mass tolerance of 20 ppm. Two missed cleavages, oxidation of methionine, C-mannosylation of tryptophan and N‑terminal acetylation were allowed.

Peptide spectrum matches were further processed by Philosopher (version 5.1) and filtered with a 1% false discovery rate (da Veiga Leprevost et al., 2020). N-glycans were searched in mass offset/ open search using the glycan module in FragPipe with the parameters FDR: 1 and glycan mass tolerance: 50 ppm (Polasky, Yu, Teo, & Nesvizhskii, 2020). Search results were visualized using FragPipe‑PDV viewer and Microsoft Excel. Obtained results were manually evaluated.

The quantification of the site-specific glycoform distribution was performed by dividing the area-under-

the-curve /AUC) of each glycoform by the summed AUC of all glycoforms identified within the same site from the same glycoprotein.

***Analyses of glycoproteins***

The algorithms Signal P 6.0 (Teufel et al., 2022) and DeepTMHMM (Hallgren et al., 2022) were used to predict signal peptide and transmembrane domain(s). The hyperplexed localisation of organelle proteins by isotope tagging and CRISPR phenotype score were retrieved from ToxoDB (Alvarez-Jarreta et al., 2024). The sequon logo was generated using WebLogo 3.7.12 version (Crooks, Hon, Chandonia, & Brenner, 2004). Multiple protein sequences alignment was carried out with MultAlin (Corpet, 1988)

**Supplementary references**

Alvarez-Jarreta, J., Amos, B., Aurrecoechea, C., Bah, S., Barba, M., Barreto, A., . . . Zheng, J. (2024). VEuPathDB: the eukaryotic pathogen, vector and host bioinformatics resource center in 2023. *Nucleic Acids Res, 52*(D1), D808-d816. doi:10.1093/nar/gkad1003

Corpet, F. (1988). Multiple sequence alignment with hierarchical clustering. *Nucleic Acids Res, 16*(22), 10881-10890. doi:10.1093/nar/16.22.10881

Crooks, G. E., Hon, G., Chandonia, J. M., & Brenner, S. E. (2004). WebLogo: a sequence logo generator. *Genome Res, 14*(6), 1188-1190. doi:10.1101/gr.849004

da Veiga Leprevost, F., Haynes, S. E., Avtonomov, D. M., Chang, H. Y., Shanmugam, A. K., Mellacheruvu, D., . . . Nesvizhskii, A. I. (2020). Philosopher: a versatile toolkit for shotgun proteomics data analysis. *Nat Methods, 17*(9), 869-870. doi:10.1038/s41592-020-0912-y

Fox, B. A., Ristuccia, J. G., Gigley, J. P., & Bzik, D. J. (2009). Efficient gene replacements in Toxoplasma gondii strains deficient for nonhomologous end joining. *Eukaryot Cell, 8*(4), 520-529. doi:10.1128/ec.00357-08

Hallgren, J., Tsirigos, K. D., Pedersen, M. D., Almagro Armenteros, J. J., Marcatili, P., Nielsen, H., . . . Winther, O. (2022). DeepTMHMM predicts alpha and beta transmembrane proteins using deep neural networks. *bioRxiv*, 2022.2004.2008.487609. doi:10.1101/2022.04.08.487609

Hütte, H. J., Tiemann, B., Shcherbakova, A., Grote, V., Hoffmann, M., Povolo, L., . . . Bakker, H. (2022). A Bacterial Mannose Binding Lectin as a Tool for the Enrichment of C- and O-Mannosylated Peptides. *Anal Chem, 94*(20), 7329-7338. doi:10.1021/acs.analchem.2c00742

Kong, A. T., Leprevost, F. V., Avtonomov, D. M., Mellacheruvu, D., & Nesvizhskii, A. I. (2017). MSFragger: ultrafast and comprehensive peptide identification in mass spectrometry-based proteomics. *Nat Methods, 14*(5), 513-520. doi:10.1038/nmeth.4256

Polasky, D. A., Yu, F., Teo, G. C., & Nesvizhskii, A. I. (2020). Fast and comprehensive N- and O-glycoproteomics analysis with MSFragger-Glyco. *Nat Methods, 17*(11), 1125-1132. doi:10.1038/s41592-020-0967-9

Teufel, F., Almagro Armenteros, J. J., Johansen, A. R., Gíslason, M. H., Pihl, S. I., Tsirigos, K. D., . . . Nielsen, H. (2022). SignalP 6.0 predicts all five types of signal peptides using protein language models. *Nature Biotechnology, 40*(7), 1023-1025. doi:10.1038/s41587-021-01156-3
