# Supplementary material for: N‐Glycoproteomics of the Apicomplexan Parasite Toxoplasma gondii
Source: Proteomics. 2025 Mar 12;25(8):e202400239. doi: 10.1002/pmic.202400239 (PMC12019905; doi:10.1002/pmic.202400239)
Supplement: Supplementary file 2 — Supporting information [file PMIC-25-e202400239-s005.pdf]

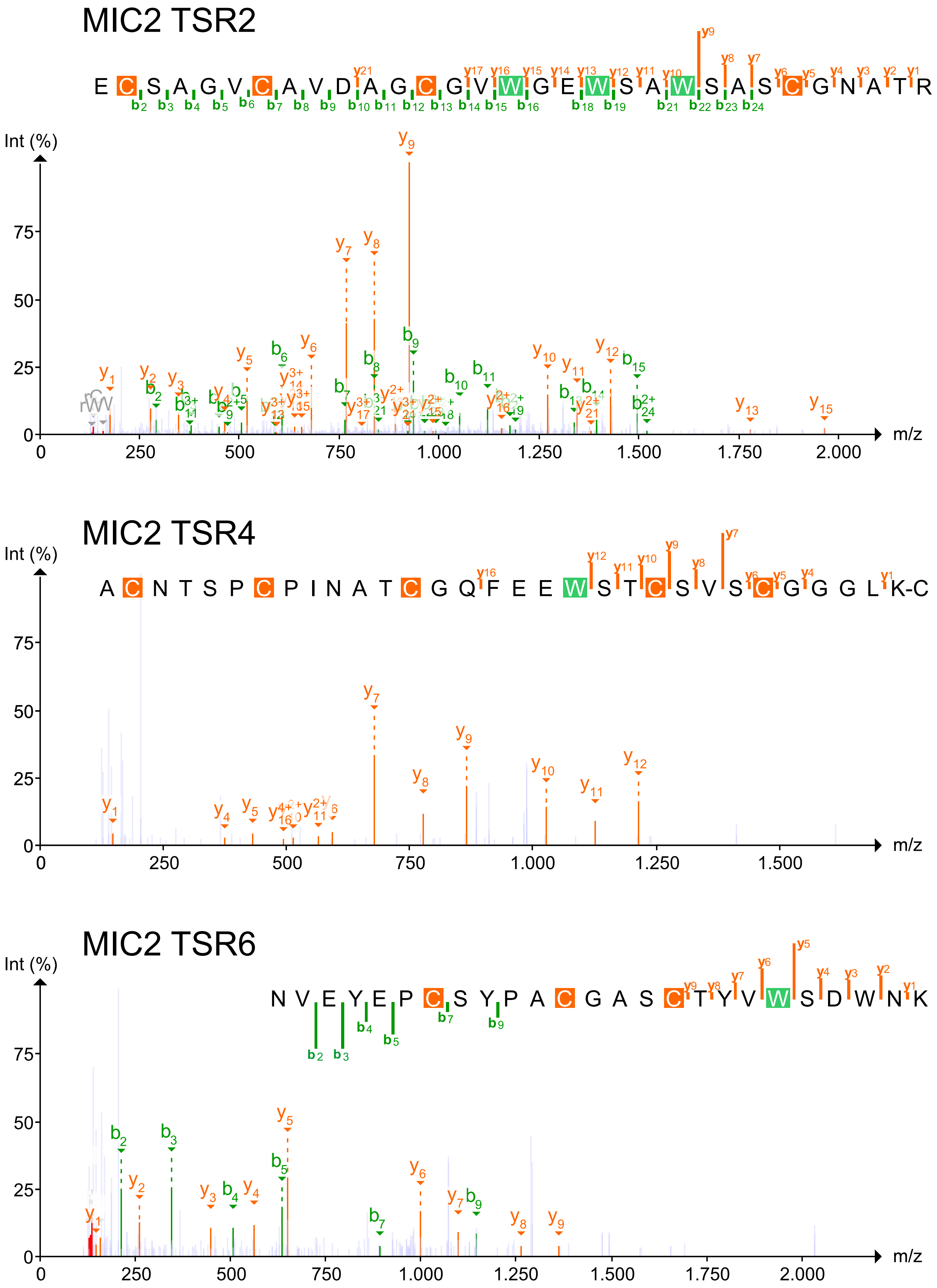

Figure S1: HCD MS/MS spectra of glycopeptides from the micromenal protein MIC2 (TGRH88_037170). TRS2 peptide ECSAGVCAVDAGCGVWGEWSAWSASCGNATR ; TSR4 peptide CNTSPCPINATCGQFEEWSTCSVSCGGGLK (spectrum E-FA_109-23_HB-362.20129) and TRS6 peptide NVEYEPCSYPACGASCTYVWSDWNK (spectrum E_FA08-24_HB416.35797). *C*-mannosylated tryptophan residues are highlighted in green.

Thrombospondin type 1 domain containing protein

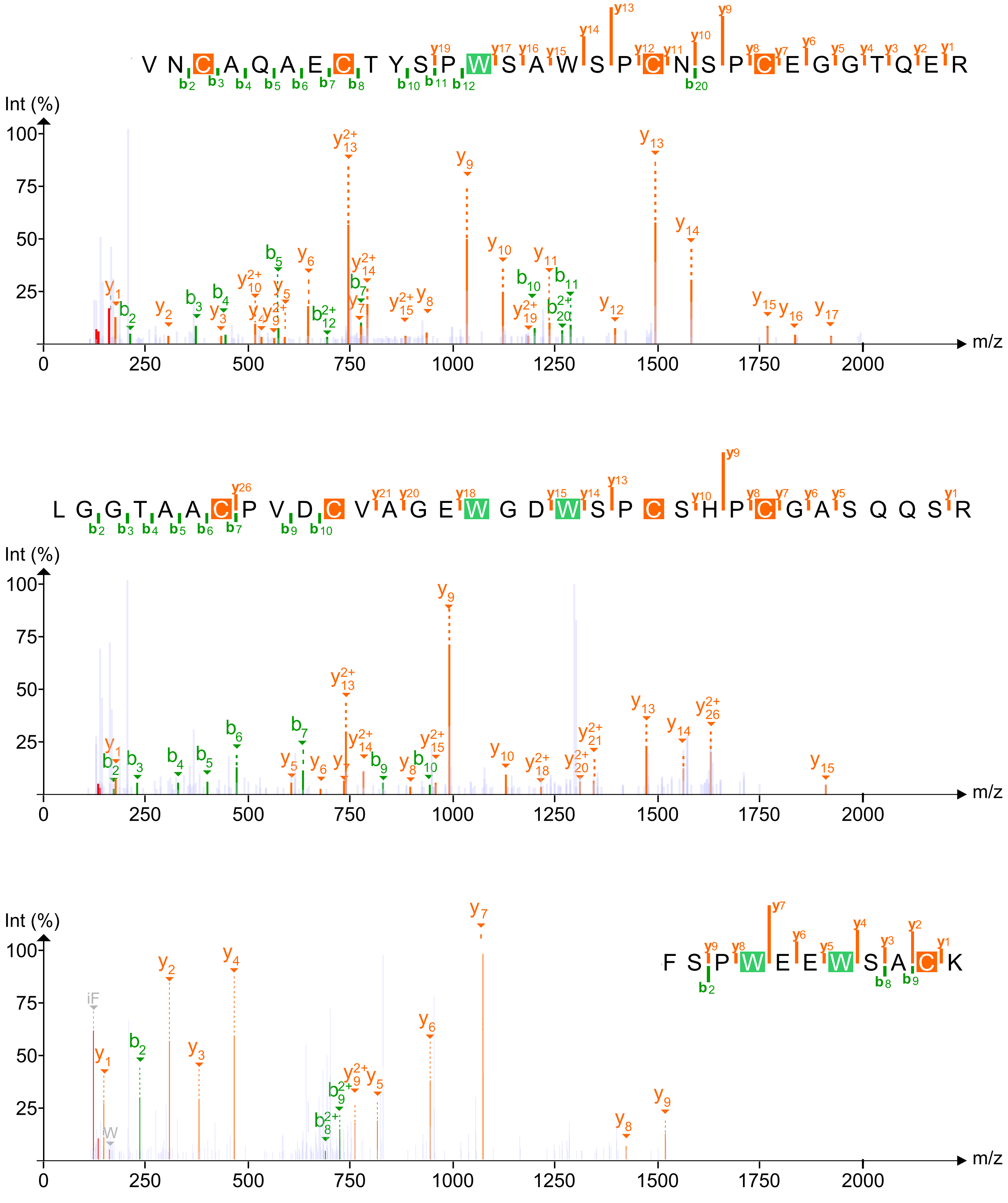

Figure S2: HCD MS/MS spectra of glycopeptides from the thrombospondin type 1 domain containing protein (TGRH88_022040). Peptide VNCAQAECTYSPWSAWSPCNSPCEGGTQER (spectrum E-FA_109-23_HB-361.28631); peptide LGGTAACPVDCVAGEWGDWSPCSHPCGASQQSR (spectrum E-FA_109-23_HB-361) and peptide DCPGFVSCGTGCVFGEWTAWSDCR (spectrum E-FA_109-23_HB-362.20970). *C*-mannosylated tryptophan residues are highlighted in green.

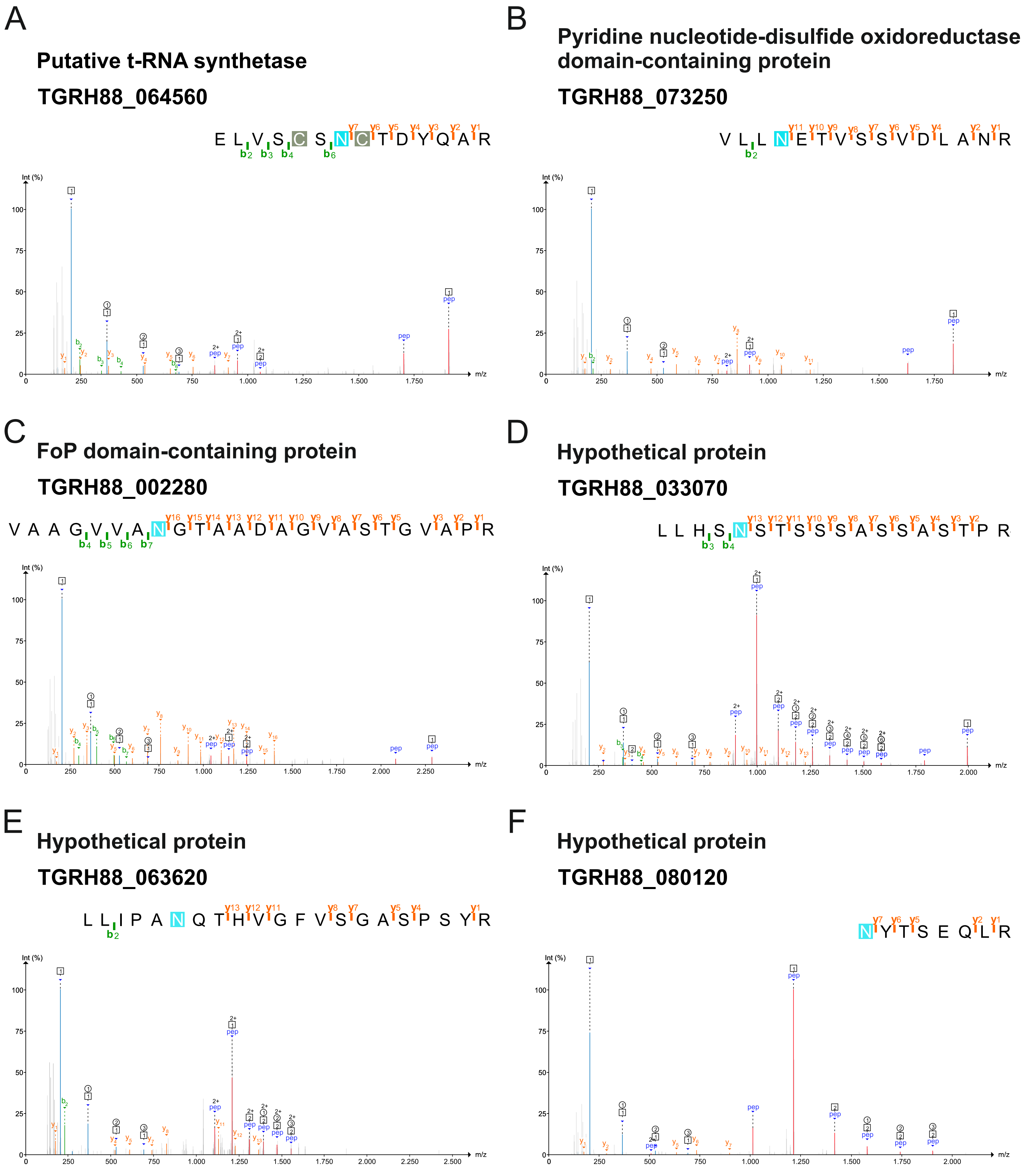

Figure S3: HCD MS/MS spectra of glycopeptides attributed to proteins predicted to localise to the cytoplasm or nucleus. A) Peptide ELVSCSNCTDYQAR of the putative serine-tRNA synthetase encoded by TGRH88_064560 with $Hex_6HexNAc_2$ N-glycan; B) Peptide VLLNETVSSVDLANR of the pyridine nucleotide-disulfide oxidoreductase domain-containing protein encoded by TGRH88_073250 with $Hex_7HexNAc_2$ N-glycan; C) Peptide VAAGVVANGTAADAGVASTGVAPR of the FoP domain containing protein encoded by TGRH88_002280 with $Hex_8HexNAc_2$ N-glycan; D) Peptide LLHSNSTSSSASSASTPR of the hypothetical protein encoded by TGRH88_033070 with $Hex_8HexNAc_2$ N-glycan; E) Peptide LIPANQTHVGFVSGASPSYR of the hypothetical protein encoded by TGRH88_063620 with $Hex_8HexNAc_2$ N-glycan; F) Peptide NYTSEQLR of the hypothetical protein encoded by TGRH88_080120 with $Hex_8HexNAc_2$ N-glycan.

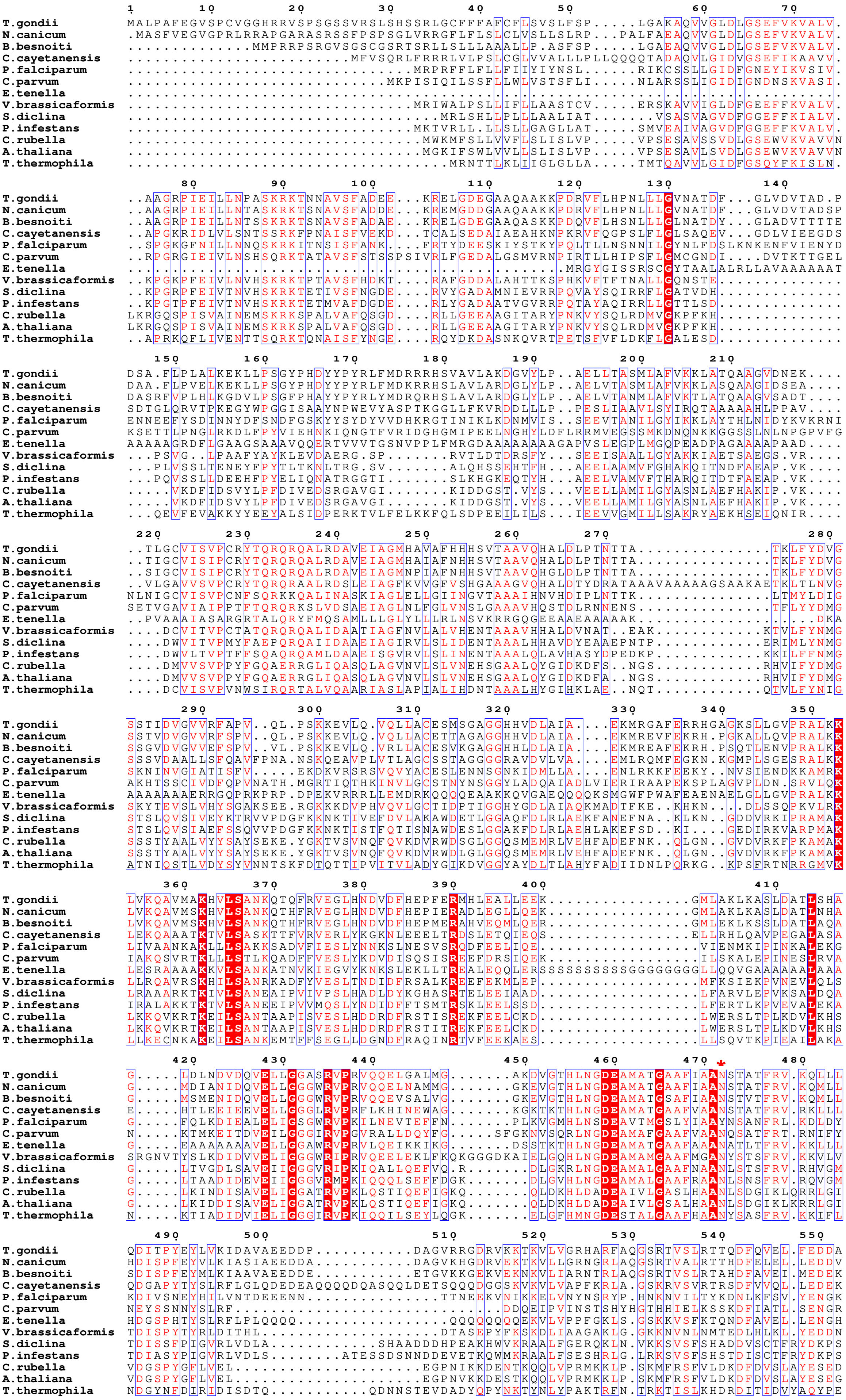
T.gondii
N.canicum
B.besnoiti
C.cayetanensis
P.falciparum
C.parvum
E.tenella
V.brassicaformis
S.diclina
P.infestans
C.rubella
A.thaliana
T.thermophila
1 10 20 30 40 50 60 70
80 90 100 110 120 130 140
150 160 170 180 190 200 210
220 230 240 250 260 270 280
290 300 310 320 330 340 350
360 370 380 390 400 410
420 430 440 450 460 470 480
490 500 510 520 530 540 550

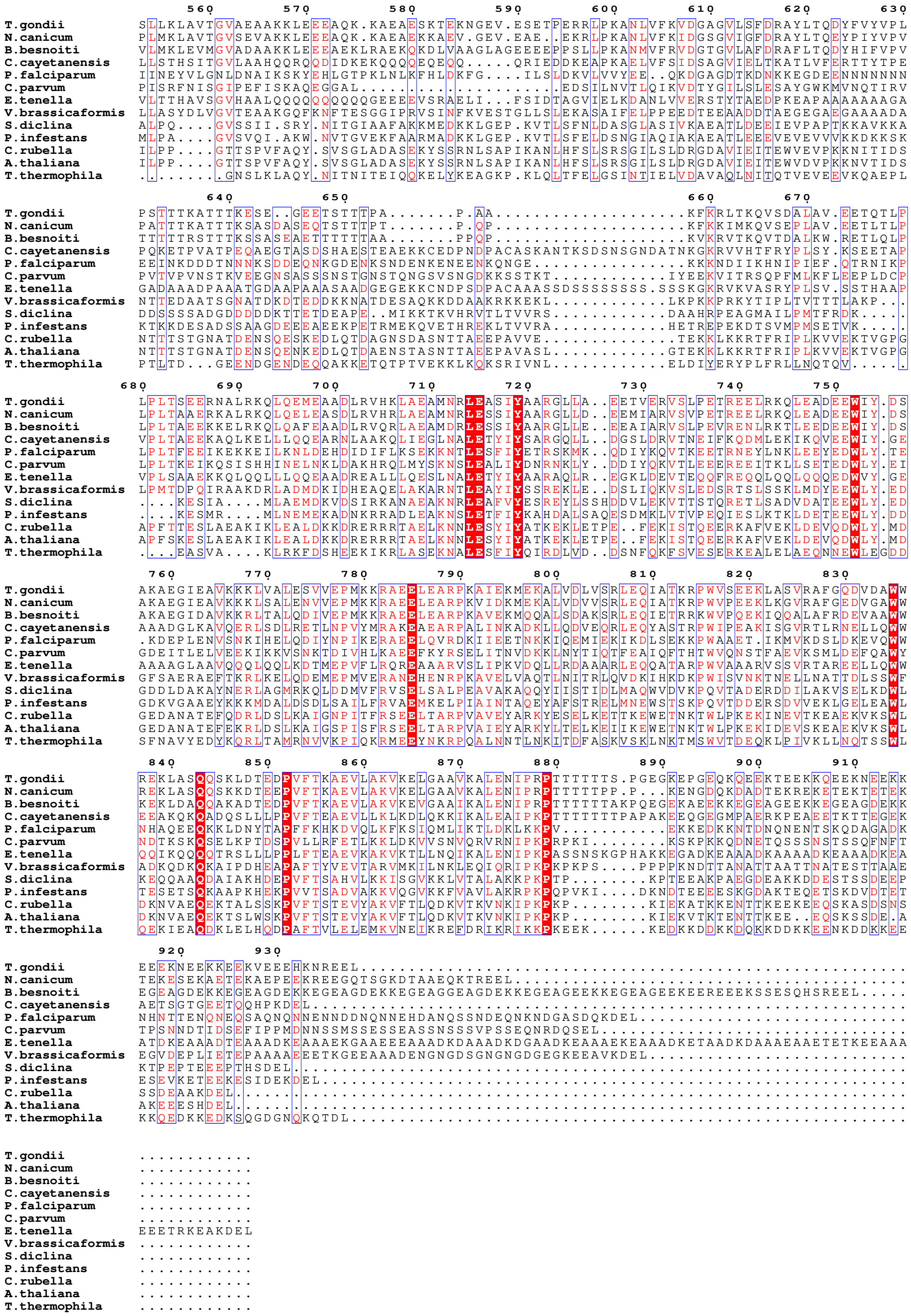

Figure S4: Alignment of *Toxoplasma gondii* putative Hsp70 (TGRH88_046410) and homologous proteins from alveolata, oomycota and plants highlighting the conservation of the glycosylation site at Asn 472. Sequences used for this alignment were from *Neospora canicum* (XP_003884216.1), *Besnoitia besnoiti* (XP_029219098.1), *Cyclospora cayetanensis* (XP_026189948.1), *Plasmodium falciparum* (XP_002809067.1), *Cryptosporidium parvum* (XP_626523.1), *Eimeria tenella* (XP_013233188.1), *Tetrahymena thermophila* (XP_001032989.2), *Vitrella brassicaformis* (CEL94101.1), *Saprolegnia diclina* (XP_008605601.1), *Phytophthora infestans* (XP_002898029.1), *Capsella rubella* (XP_006286060.1) and *Arabidopsis thaliana* (NP_567510.1). Conserved residues are highlighted in red.
